# Supplementary figures and images for: Novel Characteristics of Race-Specific Genetic Functions in Korean CADASIL
Source: Medicina (Kaunas). 2019 Aug 22;55(9):521. doi: 10.3390/medicina55090521 (PMC6780260; doi:10.3390/medicina55090521)

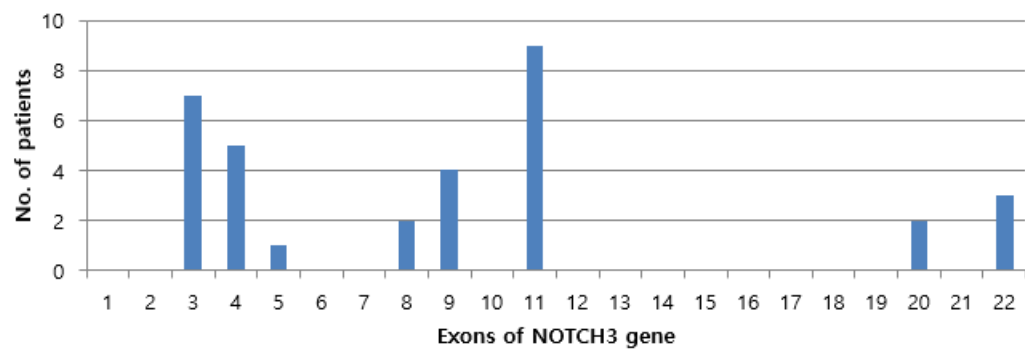

**Figure S1.** Exon distributions in NOTCH3 gene mutations.

Supplement: Supplementary file 1 [file medicina-55-00521-s001.pdf]
